# Efavirenz Has the Highest Anti-Proliferative Effect of Non-Nucleoside Reverse Transcriptase Inhibitors against Pancreatic Cancer Cells

**DOI:** 10.1371/journal.pone.0130277

**Published:** 2015-06-18

**Authors:** Markus Hecht, Sonja Erber, Thomas Harrer, Hartwig Klinker, Thomas Roth, Hans Parsch, Nora Fiebig, Rainer Fietkau, Luitpold V. Distel

**Affiliations:** 1 Department of Radiation Oncology, University Hospital Erlangen, Erlangen, Germany; 2 Department of Internal Medicine 3, University Hospital Erlangen, Erlangen, Germany; 3 Department of Internal Medicine 2, University Hospital Würzburg, Würzburg, Germany; 4 Central Laboratory, University Hospital Erlangen, Erlangen, Germany; Centro de Biología Molecular Severo Ochoa (CSIC-UAM), SPAIN

## Abstract

**Background:**

Cancer prevention and therapy in HIV-1-infected patients will play an important role in future. The non-nucleoside reverse transcriptase inhibitors (NNRTI) Efavirenz and Nevirapine are cytotoxic against cancer cells *in vitro*. As other NNRTIs have not been studied so far, all clinically used NNRTIs were tested and the *in vitro* toxic concentrations were compared to drug levels in patients to predict possible anti-cancer effects *in vivo*.

**Methods:**

Cytotoxicity was studied by Annexin-V-APC/7AAD staining and flow cytometry in the pancreatic cancer cell lines BxPC-3 and Panc-1 and confirmed by colony formation assays. The 50% effective cytotoxic concentrations (EC50) were calculated and compared to the blood levels in our patients and published data.

**Results:**

The *in vitro* EC50 of the different drugs in the BxPC-3 pancreatic cancer cells were: Efavirenz 31.5μmol/l (= 9944ng/ml), Nevirapine 239μmol/l (= 63786ng/ml), Etravirine 89.0μmol/l (= 38740ng/ml), Lersivirine 543μmol/l (= 168523ng/ml), Delavirdine 171μmol/l (= 78072ng/ml), Rilpivirine 24.4μmol/l (= 8941ng/ml). As Efavirenz and Rilpivirine had the highest cytotoxic potential and Nevirapine is frequently used in HIV-1 positive patients, the results of these three drugs were further studied in Panc-1 pancreatic cancer cells and confirmed with colony formation assays. 205 patient blood levels of Efavirenz, 127 of Rilpivirine and 31 of Nevirapine were analyzed. The mean blood level of Efavirenz was 3587ng/ml (range 162–15363ng/ml), of Rilpivirine 144ng/ml (range 0-572ng/ml) and of Nevirapine 4955ng/ml (range 1856–8697ng/ml). Blood levels from our patients and from published data had comparable Efavirenz levels to the *in vitro* toxic EC50 in about 1 to 5% of all patients.

**Conclusion:**

All studied NNRTIs were toxic against cancer cells. A low percentage of patients taking Efavirenz reached *in vitro* cytotoxic blood levels. It can be speculated that in HIV-1 positive patients having high Efavirenz blood levels pancreatic cancer incidence might be reduced. Efavirenz might be a new option in the treatment of cancer.

## Introduction

**Fig 1:**
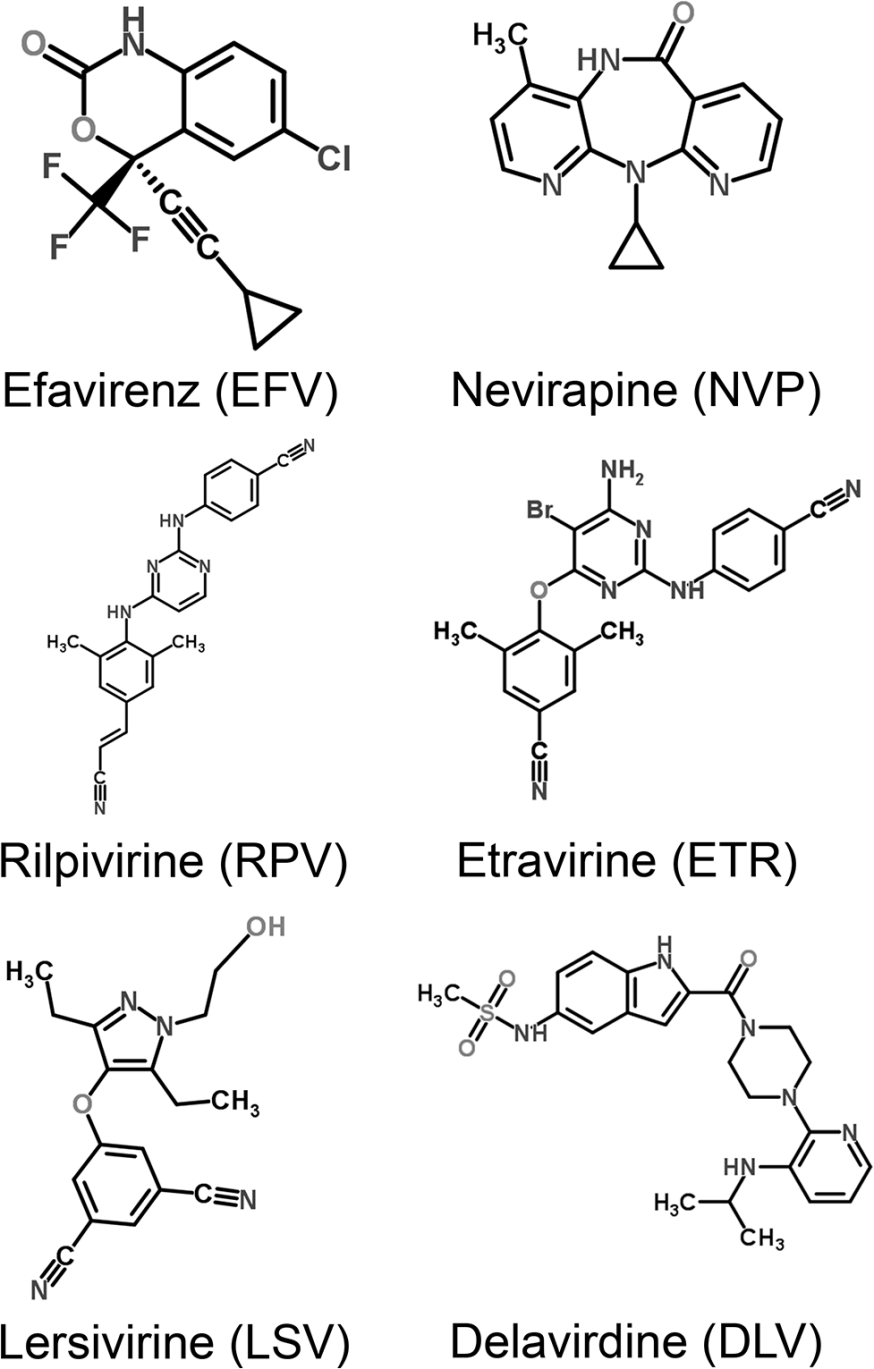
Chemical structures of the studied non-nucleoside reverse transcriptase inhibitors.

Nowadays, in HIV-1-infected patients the HIV-infection itself can be controlled very well by antiretroviral combination therapy. Consequently, life expectancy of these patients is not substantially reduced by the infection [1]. Thus, HIV-1-infected patients get older and consequently the prevention and therapy of comorbidities will play a larger role in future. As one third of all deaths in HIV-1-infected patients are cancer related, cancer prophylaxis and therapy is of prime importance [2]. In this context the data about anti-cancer effects of antiretroviral medication become increasingly important. The non-nucleoside reverse transcriptase inhibitors (NNRTI) Efavirenz (EFV) and Nevirapine (NVP) are toxic against a wide range of cancer cells *in vitro* [3–10] and only have a minor toxicity against normal tissue cells [3]. An effective cancer treatment with NNRTIs has also been proven in mice [4, 9]. As these NNRTIs are very well-tolerated in HIV treatment, they are also promising for cancer treatment. There is still no completely satisfactory scientific explanation of the mechanism of action. One explanation for the mode of operation is the inhibition of an endogenous reverse transcriptase in cancer cells [4–8], another is the interaction with the cannabinoid system [3]. Furthermore, oxidative stress in mitochondria is discussed as mechanism of action [11–13]. During the last years a new generation of NNRTIs has been developed, namely Rilpivirine (RPV), Etravirine (ETR) and Lersivirine (LSV) (Fig 1). So far, these drugs have not been tested for anti-cancer effects. Consequently, in this study EFV, NVP, RPV, ETR, LSV and Delavirdine (DLV) were investigated for toxic effects against cancer cells *in vitro*. Another crucial factor is, if the *in vitro* toxic concentrations can be reached *in vivo*. Thus, the *in vitro* toxic drug concentrations were compared to blood levels in our patients and published data. When the toxic concentrations on cancer cells can be reached *in vivo*, this might give the opportunity to treat HIV and cancer with one drug. This also might reduce the incidence of cancers in HIV-1-infected patients. Additionally, Efavirenz has a favorable long-term tolerability in HIV-1-infected patients. So it might become an option for palliative cancer treatment. It was demonstrated that it is toxic against a wide range of cancer cells [3–10]. In this study pancreatic cancer cell lines were chosen, as for patients with metastatic pancreatic cancer still exist only few and quite toxic treatment schemes.

## Materials and Methods

### Cell culture

All cell lines were cultured at 37°C in a 5% CO_2_ incubator. All cell lines were grown in Dulbecco’s Modified Eagle Medium (PAN Biotech GmbH, Aidenbach, Germany) supplemented with 10% foetal bovine serum 1% penicillin/streptomycin. The BxPC-3 cell line was obtained from the commercial source ATCC (20.2.1997, Wesel, Germany). The Panc-1 cell line was obtained from the European Collection of Cell Cultures (31.1.1997, Salisbury, Wiltshire, UK).

### Drugs

Efavirenz (EFV), Nevirapine (NVP), Rilpivirine (RPV), Etravirine (ETR), Lersivirine (LSV) ahd Delavirdine (DLV) (Sequoia Research Products Limited, Pangbourne, UK) were dissolved in DMSO as 10 mmol/l stock solutions. The toxicity against cancer cells was tested in a concentration range from one to 1000μmol/l.

### Flow cytometry

Apoptosis and necrosis was detected by the Annexin-V-APC (Annexin-V-Allophycocyanin) and 7AAD (7-Aminoactinomycin) assay (BD Pharmingen, Franklin Lakes, USA). Briefly, cells were suspended in Ringer solution and stained with Annexin-V-APC (dilution 1:40) and 7AAD (dilution 1:40) for 30min at 4°C. Each run 10.000 cells were analyzed. A Gallios flow cytometer (Gallios Cytometer 1.1 Software Beckmann Coulter, Krefeld, Germany) was used. Results were analyzed with Kaluza Flow Cytometry Analysis 1.1 (Beckmann Coulter, Krefeld, Germany). Experiments were performed three times with three replicates per run.

### Colony formation assay

The different drugs were added 24h after cell seeding. Medium containing the drug was removed after an incubation period of 72h. The cultures were incubated for three weeks at 37°C. Colonies were stained with methylene blue and clusters containing 50 or more cells were scored as a colony. Analyses were done computer assisted to evaluate the number of the colonies [14]. Results were calculated as Survival fraction (SF). Experiments were performed three times with three wells per experiment.

### Patients

All EFV and NVP blood levels measured between 2009 and 2014 in the Central Laboratory at the University Hospital Erlangen and all RPV drug levels measured between 2013 and 2015 at the laboratory of the University Hospital Würzburg were retrospectively analyzed. Approval was given by the Ethics Committee of the Medical Faculty of the Friedrich-Alexander-University of Erlangen-Nürnberg (Number: 3376) and the Ethics Committee of the Medical Faculty of the Julius-Maximilian-University of Würzburg (Number: F-204). Informed consent was obtained from all patients in written form. The blood levels were determined in freshly drawn blood samples using high performance liquid chromatography (HPLC). Patient records were anonymized.

### Statistics

Graphics were plotted using TechPlot 7 (SFTek, Braunschweig, Germany). Graphics were fitted for the fraction of dead cells in the Annexin-V-APC/AAD assay and the surviving fraction in the colony formation assay. All data were fitted according to:

$$y=\frac{x^{p0}}{p1+x^{p0}}\;\text{or}\;y=1-\frac{x^{p0}}{p1+x^{p0}}$$

The parameters p0 and p1 were calculated by least square approximation. The 50% effective concentration (EC50) was estimated from the fitted function values.

## Results

### High toxic potential of Efavirenz and Rilpivirine against cancer cells

**Fig 2:**
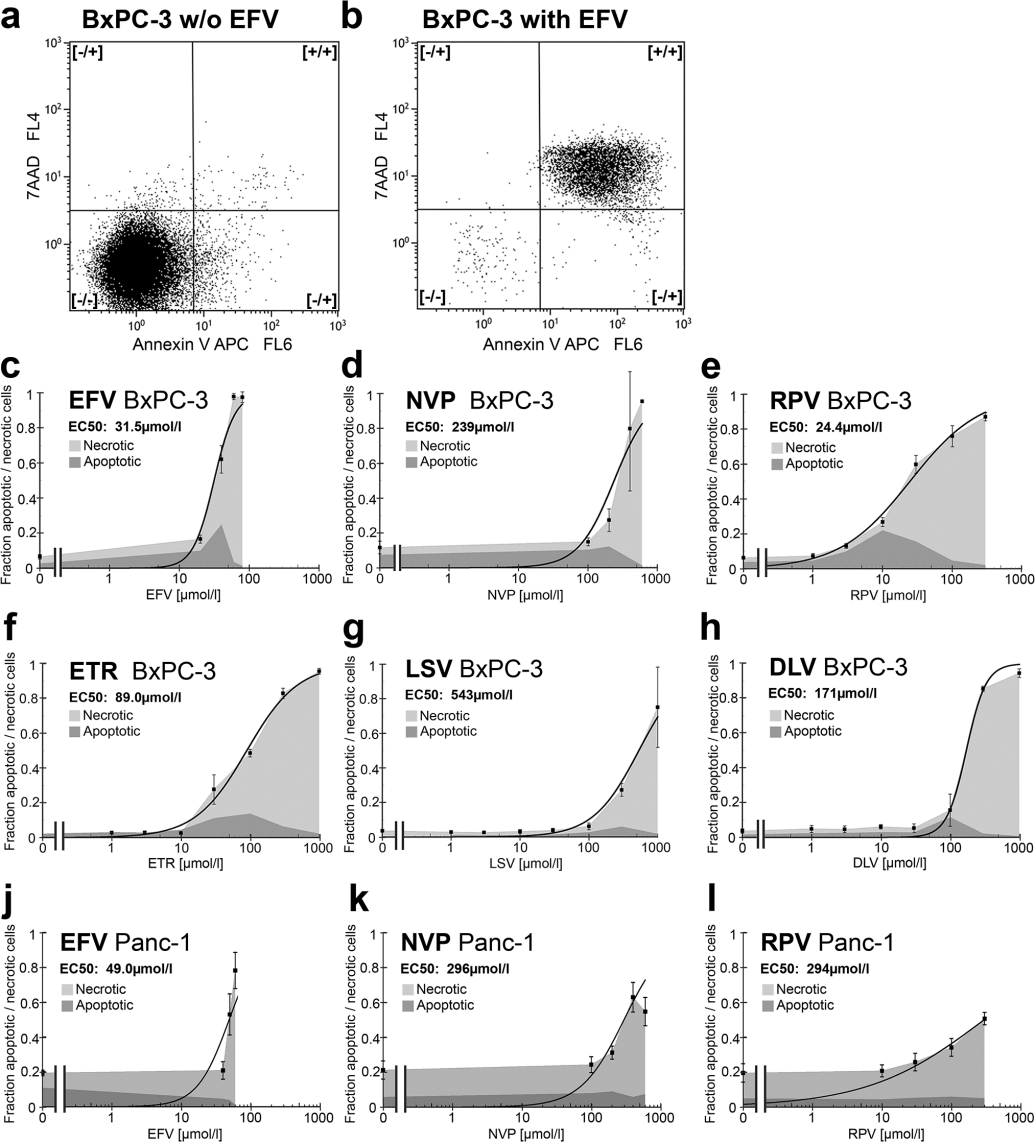
Apoptosis and necrosis induction in cancer cells by non-nucleoside reverse transcriptase inhibitors. The fraction of apoptotic and necrotic cells after treatment with different NNRTIs in different concentrations was measured by Annexin-V-APC/7AAD staining and flow cytometry. An example of the gating in the FACS plots is shown for untreated (a) and with a toxic concentration of EFV treated cells (b). A curve was fitted through the data points of the total fraction of dead cells and the EC50 was calculated for each drug. The pancreatic cancer cell line BxPC-3 was treated for 72h with (c) EFV, (d) NVP, (e) RPV, (f) ETR, (g) LSV and (h) DLV. The pancreatic cancer cell line Panc-1 was treated for 72h with (j) EFV, (k) NVP and (l) RPV.

Published data report a toxic effect of EFV and NVP against cancer cells, whereas EFV is toxic at lower concentrations than NVP [4, 5, 7]. As a new generation of NNRTI has been developed, the question raised whether these drugs are also toxic against cancer. So in the following the NNRTIs EFV, NVP, RPV, ETR, LSV and DLV were studied for toxic effects against BxPC-3 pancreatic cancer cells *in vitro* (Fig 1). The cells were treated with the different drugs for 72h. The potential of these drugs to induce apoptosis and necrosis was analyzed by Annexin-V-APC/7AAD staining and flow cytometry. Annexin-V-APC/7AAD double negative cells were considered as viable cells, Annexin-V-APC-positive/7AAD-negative cells were considered as apoptotic cells and Annexin-V-APC/7AAD double-positive cells were considered as necrotic cells [15] (Fig 2A and 2B). A function was fitted to the data of the total amount of dead cells and the 50% effective concentration (EC50) was calculated (Fig 2C–2H). All NNRTIs are toxic against cancer cells, whereas at lower doses apoptosis and at higher doses necrosis is the leading type of death. But the toxic concentrations of the different drugs differ widely. RPV and EFV are toxic already at low concentrations (EC50: RPV 24.4μmol/l, EFV 31.5μmol/l). ETR is only toxic at three fold higher concentrations (EC50: 89.0μmol/l). NVP, DLV and LSV become not toxic up to six fold the toxic doses of EFV or RPV (EC50: NVP 239μmol/l, DLV 171μmol/l, LSV 543μmol/l). The toxicity of EFV arises steeply when a dose limit is exceeded. In contrast, the toxicity of RPV rises slowly with increasing drug concentrations. These results were confirmed in Panc-1 pancreatic cancer cells for the most toxic agents EFV and RPV. Additionally for the less toxic, but in vivo frequently used NVP was tested. In this cell line EFV had a higher toxicity (EC50: 49.0μmol/l) than NVP and RPV (EC50: 296μmol/l and 294μmol/l) (Fig 2J–2L). Thus only EFV has a toxicity at low concentration against both pancreatic cancer cell lines.

**Fig 3:**
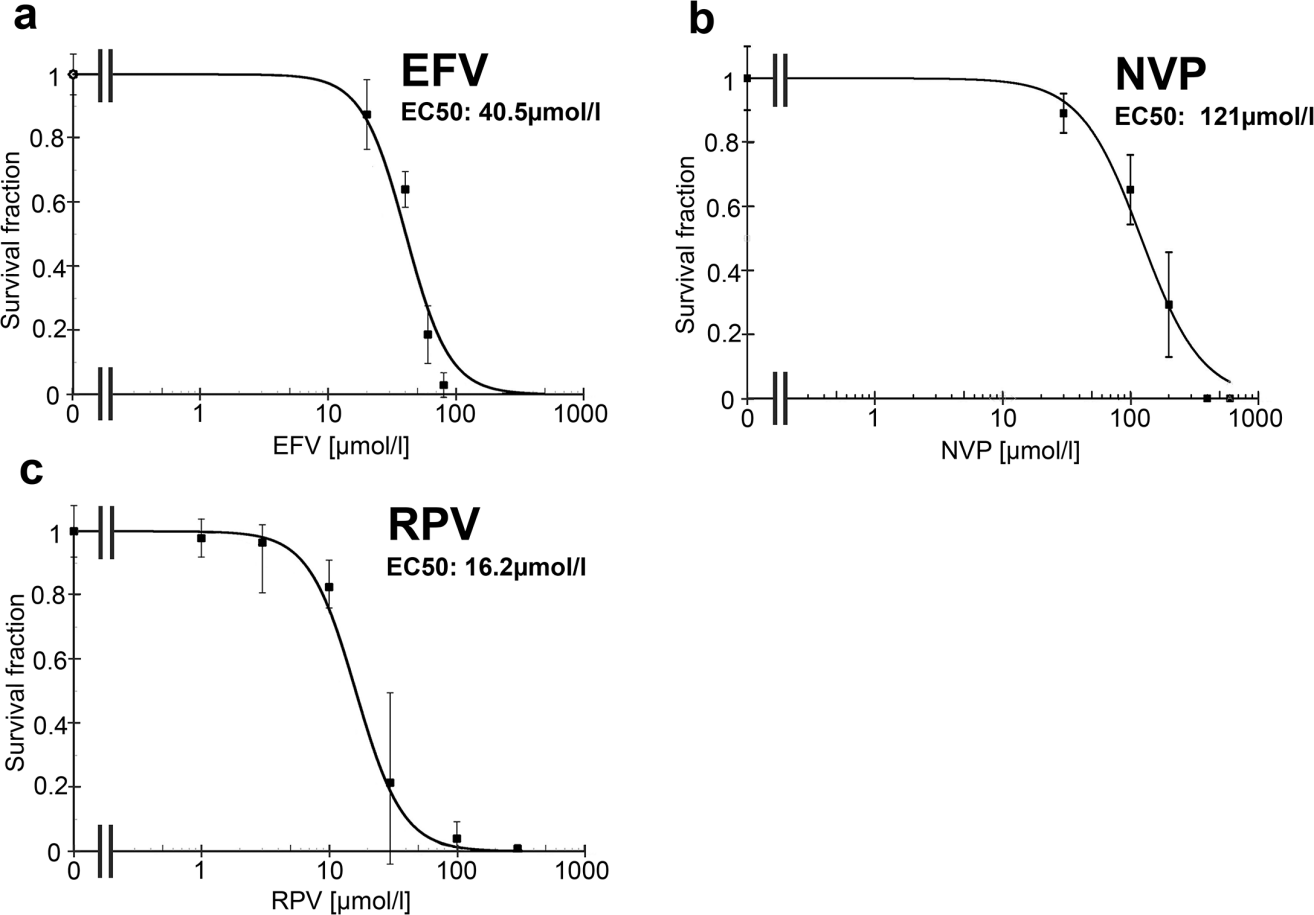
Effect of EFV, NVP and RPV on the survival fraction of cancer cells. Colony formation assays were performed with (a) EFV, (b) NVP and (c) RPV. The pancreatic cancer cell line BxPC-3 was treated for 72h with each of the drugs. The survival fraction (SF) was analyzed and normalized to the control. Graphs were fitted and the EC50 was calculated.

As further confirmation of these results, the toxicity of EFV, NVP and RPV was studied with colony formation assays of BxPC-3 pancreatic cancer cells (Fig 3). In the colony formation assays the survival fraction (EC50) of EFV (40μmol/l) and RPV (16.2μmol/l) were lower than the EC50 of NVP (121μmol/l). This corresponds to the Annexin-V-APC/7AAD staining. EFV and RPV are clearly toxic against BxPC-3 pancreatic cancer cells at lower concentrations compared to other NNRTIs *in vitro*.

### *In vitro* toxic concentrations of Efavirenz against tumor can be reached in a low percentage of patients

These results raise the question whether EFV or RPV can be used as anti-cancer drug in patients. The key point of this question is, if the *in vitro* toxic drug concentrations can be reached *in vivo*. Consequently we analyzed drug levels in the blood of HIV-1-infected patients.

In the Central Laboratory of the University Hospital Erlangen blood levels of EFV and NVP are analyzed in routine diagnostic. Altogether 205 blood levels of EFV and 31 blood levels of NVP were determined between 2009 and 2014 using high performance liquid chromatography (HPLC). Furthermore, 127 blood levels of RPV were analyzed with HPLC at the laboratory of the University Hospital Würzburg between 2013 and 2015. Efavirenz serum levels were measured in blood samples usually obtained 8 to 13 hours after taking the drug, whereas RPV and NVP serum levels measurements were performed at various time points after intake.

**Fig 4:**
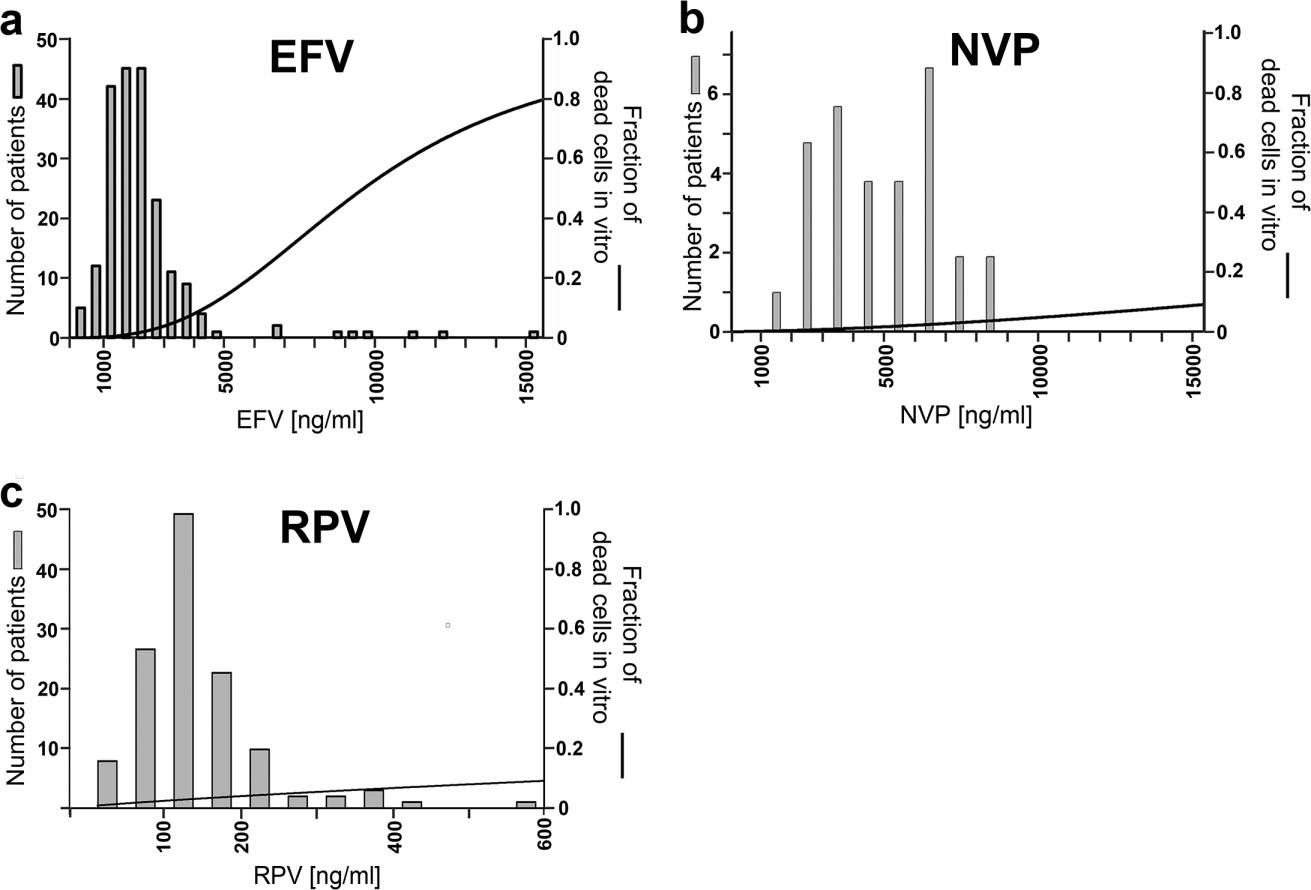
Comparison of blood levels in patients with in vitro toxic EC50 of EFV, NVP and RPV. Blood levels of (a) EFV, (b) NVP and (c) RPV determined by HPLC (bars). These in vivo concentrations are compared to the fitted function of the in vitro toxicity against BxPC-3 pancreatic cancer cells in the Annexin-V-APC/AAD staining (solid line).

The mean EFV level in our patients was 3587ng/ml (equivalent 11.4μmol/l). The median was 2055ng/ml (equivalent 6.51μmol/l) with a total range from 162 to 15363ng/ml (equivalent 0.513 to 48.7μmol/l) (Fig 4A). When these blood levels are compared to the *in vitro* toxic doses, 3 levels (1.5%) were above the in vitro toxic EC50 of 31.5μmol/l (Annexin-V-APC/7AAD staining, BxPC-3). It can be concluded that *in vitro* the anti-cancer effective concentrations of EFV are close to the doses in vivo and can be reached in a low percentage of patients.

The mean NVP level was 4955ng/ml (equivalent 18.6μmol/l). The median was 4992ng/ml (equivalent 18.7μmol/l) with a total range from 1856 to 8697ng/ml (equivalent 6.95 to 32.6μmol/l) (Fig 4B). All these blood levels were far below the *in vitro* toxic concentrations (EC50) for apoptosis/necrosis.

The mean RPV level was 144ng/ml (equivalent 0.39μmol/l). The median was 128ng/ml (equivalent 0.35μmol/l) with a total range from 0 to 572ng/ml (equivalent 0 to 1.56μmol/l) (Fig 4C). When these blood levels are compared to the *in vitro* toxic concentrations, none reached the EC50 for apoptosis/necrosis. Thus, the concentrations of NVP and RPV in patient’s blood are much lower than the cytotoxic concentration against cancer cells *in vitro*. Only single patients taking EFV reach the *in vitro* toxic EC50.

## Discussion

Cytotoxic effects of EFV and NVP against cancer cell lines and cancers in animal models have been reported several times. However, the anti-cancer activity of NVP is approximately ten fold lower than the one of EFV [3–10]. During the last years, new NNRTIs have been developed and applied in clinical practice. An anti-cancer cell activity of the other NNRTIs DLV, ETR, RPV or LSV has not been studied so far. We could show that all NNRTIs are toxic against cancer cells with a broad range of toxic concentrations. RPV and EFV are toxic against pancreatic cancer cells at lowest concentrations. We found EC50s of EFV (31.5μmol/l and 49.0μmol/l) which are comparable to published concentrations that show anti-cancer cell activity *in vitro* (range 10–60μmol/l) [3–5, 7, 11–13]. In BxPC-3 cells the lower toxic NNRTI concentrations induced apoptosis and the higher concentrations necrosis, whereas in the Panc-1 cells no increase of apoptosis was found. Furthermore, the Panc-1 cells were altogether more resistant to NNRTI treatment. Besides several minor differences of the two cell lines, BxPC-3 harbors wild-type K-RAS, whereas in Panc-1 K-RAS is mutated [16]. This might be a reason for the different sensitivity to NNRTI treatment and should be considered in future studies focusing the mechanism of action.

The toxicity of NNRTIs against cancer cells promotes the idea to use these drugs in HIV-1-infected patients to prevent or even treat cancer. But the crucial factor is, whether the *in vitro* toxic concentrations can be achieved *in vivo*. In our patients the *in vitro* toxic EC50 of EFV was reached in 1.5% of all quantified samples, whereas none of the patients taking RPV or NVP reached the *in vitro* toxic EC50. In Table 1 published NNRTI blood levels in patients are compared to the *in vitro* toxic concentrations against cancer cells. If a study contained more patient groups, the group with the highest dose was chosen. Even without escalating the common dose, a low percentage of patients taking EFV reached the *in vitro* toxic EC50 (2.4–5.2%). These results are comparable to the results in our patients (1.5%). None of the patients, who received other NNRTIs, reached the respective EC50. In one study the EFV dose was escalated to 800mg due to a combination with the cytochrome P450 inducer rifampicin. Consequently 14.3% of these patients achieved the *in vitro* toxic EC50. As rifampicin reduces the EFV concentrations by cytochrome P450 induction, 800mg EFV without Rifampicin might even lead to higher EFV blood concentrations [17]. Furthermore, the mean of the blood concentrations of all patients was compared to the EC50 of the different drugs. The *in vitro* EC50 of EFV was approximately three times as high as the levels measured in patients, which is comparable with the result of our patients (increase by a factor of 2.8). Among the other NNRTIs larger differences of the *in vitro* toxic EC50 and the patient’s blood levels were found (Table 1). All these blood levels were quantified approximately 12h after drug intake. Thus the initial drug levels were higher than the measured values, because the maximal blood concentration is achieved approximately 3h after drug intake [18]. In this context, it also has to be considered that the treatment time of the cell line *in vitro* was 72h. A continuous treatment *in vivo* might even be more toxic against cancer cells.

**Table 1:** Blood levels of various NNRTIs.

Drug *(in vitro EC 50)*	Author	Daily Dose	Limitations	Measured after intake	Measured samples	C mean (ng/ml)	C max (ng/ml)	Number of blood levels > EC50	Ratio EC50 / C mean
**EFV**	Manosuthi 2005 [19]	1x600mg	concurrent Rifampicin	12h	42	3020	12210	1 (2.4%)	3.3
*(31*.*5μmol/l* =	“	1x800mg	concurrent Rifampicin	12h	42	3390	21310	6 (14.3%)	2.9
*9944ng/ml)*	Marzolini 2001 [20]	1x600mg		14h	171	2188	15230	6 (3.5%)	4.5
	Gutierrez 2005 [21]	1x600mg		12h	58	4120	12590	3 (5.2%)	2.4
**NVP**	Ratanasuwan 2012 [22]	2x200mg		Post-dose	108	6671	~25000	0	9.6
*(239μmol/l* =	Nafrialdi 2012 [23]	2x200mg			24	7950	~15000	0	8.0
*63786ng/ml)*	Wang 2011 [17]	2x200mg	CR group	2h	87	6850	~15000	0	9.3
	“	2x200mg	CR group	12h	34	5180			12.3
**ETR**	DeJesus 2010 [24]	1x400mg		4h	20	801	1410	0	48.4
*(89*.*0μmol/l* =	Kakuda 2009 [25]	2x800mg	early formulation	3h	15	935			41.5
*38740ng/ml)*	Gruzdev 2003 [26]	2x900mg	early formulation	3h	12	418			92.7
**LSV**	Vourvahis 2012 [27]	1x2400mg	healthy volunteers	4h	48	1727	~3000	0	97.6
*(543μmol/l* =	Vourvahis 2010 [28]	1x750mg	healthy volunteers	4h	14	1524			111
*168523ng/ml)*	Vourvahis 2012 [29]	2x1000mg	healthy volunteers	4h	16	1328			127
**DLV** *(171μmol/l*	Justesen 2004 [30]	2x1000mg	healthy volunteers	1h	3	14890	24840	0	5.2
= *78072ng/ml)*	Cheng 1997 [31]	3x400mg		1h	8	12144			6.4
**RPV** *(24*.*4μmol/l*	Goebel 2006 [32]	1x150mg		4h	9	922			9.7
= *8941ng/ml)*	Ford 2013 [33]	1x25mg	healthy volunteers	4h	16	148			60.4

Several studies about blood concentrations of NNRTIs in patients are compared to the in vitro EC50 of cytotoxicity against cancer cells. (C mean: mean of all measured blood concentrations in a cohort; C max: highest blood concentration measured in one patient in a cohort)

In this study the toxic effect of the different NNRTIs was exclusively studied in two pancreatic cancer cell lines. Nevertheless, EFV is toxic against a large range of different cancer cell lines *in vitro* including colorectal carcinoma, head and neck squamous cell carcinoma, glioblastoma, lymphatic cancer, renal carcinoma, prostate carcinoma, melanoma, small cell lung carcinoma and thyroid carcinoma [3–5, 7].

But it has to be clarified, that this study only compares the *in vitro* toxicity to blood levels of HIV-1 positive patients. The EC50 is an established value to compare toxicity. But it does not prove, that if blood concentrations are rising up to the level of the *in vitro* EC50, an anticancer effect will be observed. As no clinical trial exists so far, the results of this comparable study support the use of EFV in future clinical trials with NNRTIs in cancer patients.

There exist different theories about the mechanism of NNRTI’s toxicity against cancer cells. One theory is the inhibition of an endogenous reverse transcriptase, which is only activated in cancer cells and essential for their malignancy [4–9]. In this context the comparison of the anti-retroviral potential of the different NNRTIs in wild type HIV-1 isolates with the cytotoxic potential against cancer cells is highly interesting. RPV inhibits HI-viruses at lowest concentrations, followed by EFV, ETR, DLV, NVP and LSV [34–36]. This reflects the order of the toxic potential against BxPC-3 pancreatic cancer cells, which was detected in this study. These finding supports the theory that the target is an endogenous reverse transcriptase in cancer cells, which is very similar to the reverse transcriptase of the HI-virus. On the other hand, the large differences of the toxic concentrations against cancer cells and the differing toxicity of RPV, but not EFV, in the two pancreatic cancer cell lines, argues against this theory and supports different mechanisms of action. Furthermore, in previously published cloning experiments the suspected reverse transcriptase of cancer cells ORF2 was not inhibited by NNRTIs, which argues against this theory [37, 38].

Another research group studied EFV’s liver toxicity and found that EFV causes oxidative stress and mitochondrial damage, which leads to apoptosis in liver cells [11–13]. As cancer cells consume more energy and contain more mitochondria than normal tissue, mitochondrial toxicity of EFV might also be an important step in the mechanism [39].

As mentioned in the introduction life expectancy of HIV-1-infected patients is prolonged and cancer becomes more frequent in aging HIV-1-infected patients. In large epidemiological studies patients on HAART had a reduced risk also of non-aids-defining cancers, which might be a hint for an anti-cancer activity of some drugs used in HAART [40, 41]. In one analysis the cancer incidence of patients without HAART did not differ from patients on NNRTI based HAART or protease inhibitor based HAART [42]. Merely this study did not distinguish between patients taking EFV and patients on NVP, which was much less effective in our analysis. But there exist epidemiologic data about regressions of precancerous cervical lesions in HIV-infected women when antiretroviral therapy was started [43, 44].

The dose calculations in this study showed that the *in vitro* toxic concentrations of EFV against cancer cells can be achieved *in vivo* in a low percentage of patients without increasing the standard dose of 600mg EFV daily. So far there exists only one clinical trial in which NNRTIs were used to treat cancer in HIV-negative patients [45]. Fifty-three patients with metastatic castration-resistant prostate cancer were treated with EFV 600mg once daily. The overall PSA (prostate specific antigen) progression rate at three months was 72%. Interestingly, the subgroup of patients with EFV plasma levels above 3000ng/ml only had a PSA progression rate of 28%. These finding supports our result, that a low percentage of patients taking EFV reach effective anti-cancer blood levels. Also in thyroid cancers an anti-cancer activity of NNRTIs has been described in HIV-negative patients [46, 47]. In addition there exist case reports about the regression of lymphomas under NNRTI based HAART and one case of a long-term survival of a patient with a small cell lung cancer [48–50].

These data confirm the idea, that EFV could be used as cytotoxic drug against cancer also *in vivo*. In HIV-1-infected patients taking EFV a reduced incidence of pancreatic cancers might be achieved. Furthermore, Efavirenz might lead to a delayed progression or even tumor shrinkage in patients with cancers.

## Conclusion

Efavirenz is the only NNRTI that has the potential to be used for cancer treatment. It can be speculated, that in patients having high Efavirenz blood levels pancreatic cancer incidence might be reduced. Efavirenz might be a new option in the treatment of cancer.
